# The nuclease-associated short prokaryotic Argonaute system nonspecifically degrades DNA upon activation by target recognition

**DOI:** 10.1093/nar/gkad1145

**Published:** 2023-12-04

**Authors:** Xueling Lu, Jun Xiao, Longfei Wang, Bin Zhu, Fengtao Huang

**Affiliations:** Key Laboratory of Molecular Biophysics, the Ministry of Education, College of Life Science and Technology, Huazhong University of Science and Technology, Wuhan, Hubei 430074, China; School of Pharmaceutical Sciences, Wuhan University, Wuhan, 430072, China; School of Pharmaceutical Sciences, Wuhan University, Wuhan, 430072, China; Key Laboratory of Molecular Biophysics, the Ministry of Education, College of Life Science and Technology, Huazhong University of Science and Technology, Wuhan, Hubei 430074, China; Shenzhen Huazhong University of Science and Technology Research Institute, Shenzhen 518063, China; Key Laboratory of Molecular Biophysics, the Ministry of Education, College of Life Science and Technology, Huazhong University of Science and Technology, Wuhan, Hubei 430074, China

## Abstract

Prokaryotic Argonautes (pAgos) play a vital role in host defense by utilizing short nucleic acid guides to recognize and target complementary nucleic acids. Despite being the majority of pAgos, short pAgos have only recently received attention. Short pAgos are often associated with proteins containing an APAZ domain and a nuclease domain including DUF4365, SMEK, or HNH domain. In contrast to long pAgos that specifically cleave the target DNA, our study demonstrates that the short pAgo from *Thermocrispum municipal*, along with its associated DUF4365-APAZ protein, forms a heterodimeric complex. Upon RNA-guided target DNA recognition, this complex is activated to nonspecifically cleave DNA. Additionally, we found that the TmuRE-Ago complex shows a preference for 5′-OH guide RNA, specifically requires a uridine nucleotide at the 5′ end of the guide RNA, and is sensitive to single-nucleotide mismatches between the guide RNA and target DNA. Based on its catalytic properties, our study has established a novel nucleic acid detection method and demonstrated its feasibility. This study not only expands our understanding of the defense mechanism employed by short pAgo systems but also suggests their potential applications in nucleic acid detection.

## Graphical Abstract

**Graphical Abstract:**
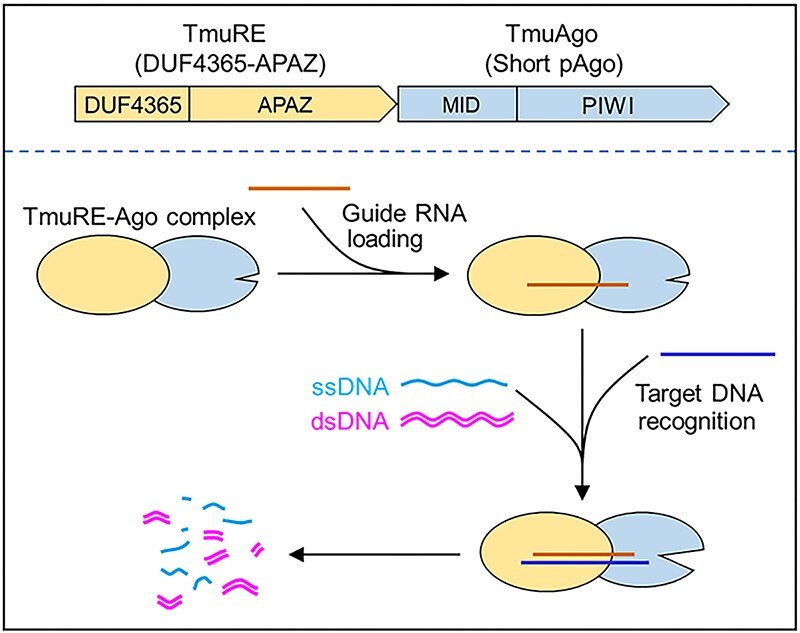

## Introduction

**Figure 1.**
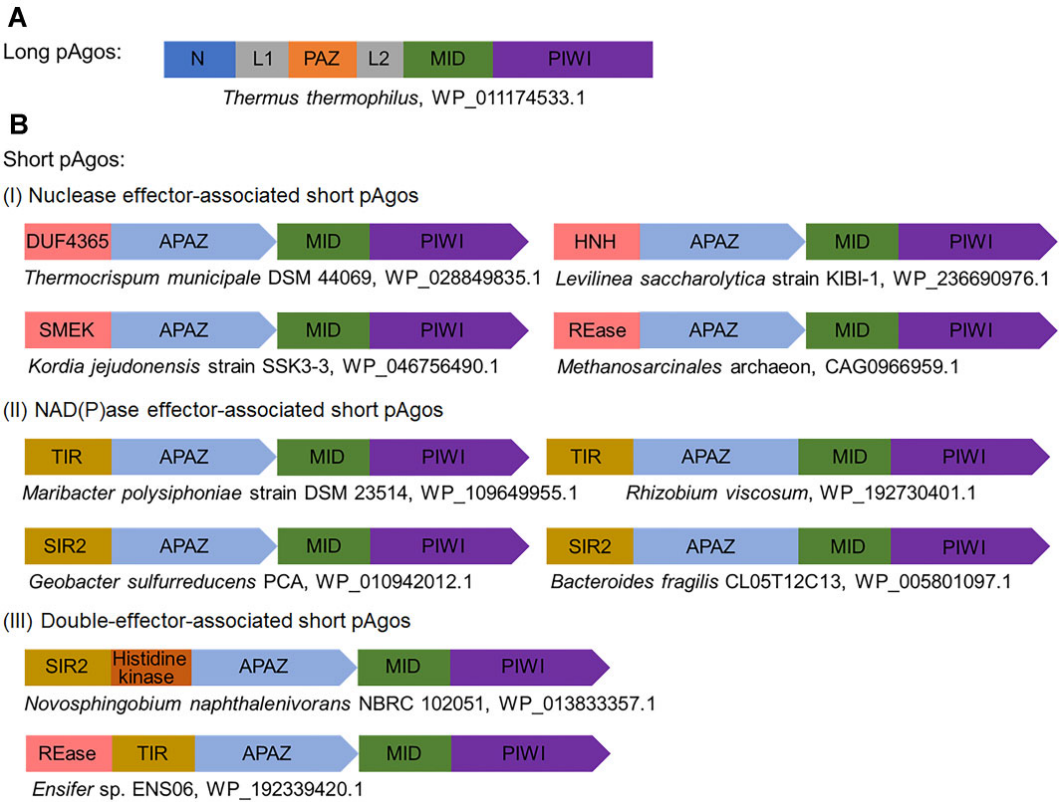
Representative domain architectures of long pAgo, and short pAgo systems. (**A**) Domain architecture of a long pAgo from *Thermus thermophilus*. (**B**) Summarization and classification of short pAgos and their associated APAZ proteins. Short pAgo systems are divided into three groups according to the APAZ proteins fused with different effector domains.

Argonaute proteins were first identified in eukaryotes as essential players in RNA interference (RNAi) pathways. Eukaryotic Argonautes (eAgos) bind to small RNA guides and use them to locate and cleave complementary RNA targets. Homologous prokaryotic Argonautes (pAgos) are found extensively in both bacteria and archaea, and they are more diverse than eAgos. pAgos can be classified into long pAgos (including long-A pAgos and long-B pAgos) and short pAgos (1,2). Long pAgos share a similar domain architecture to eAgos, including N (N-terminal), PAZ (PIWI-Argonaute-Zwille), MID (middle), and PIWI (P element-induced wimpy testis) domains (Figure 1A). The PIWI domain adopts an RNase H fold, which contributes to target cleavage. The PAZ and MID domains are involved in binding the 3′-end and 5′-end of the guide oligonucleotides, respectively, while the N domain is proposed to facilitate the dissociation of the cleaved target DNA or RNA (2,3). Several long-A pAgos have been characterized, and most prefer to utilize single-stranded (ss)DNA guides to cleave complementary ssDNA targets (4–7). Recently, some long-A pAgos were found to use ssDNA guides to cleave complementary RNA targets (8–10). The nucleic acid-guided cleavage activities of long-A pAgos are critical for defense against invading nucleic acids, such as plasmids and viruses (4,11). Unlike long-A pAgos, long-B pAgos utilize RNA guides to bind DNA targets, but they cannot cleave the targets due to the absence of the catalytic DEDX tetrad (12). A recent study showed that long-B pAgos, in conjunction with their associated effector proteins including nuclease, SIR2-domain-containing proteins and transmembrane proteins, play a role in host defense by inducing abortive infection (13). While our knowledge of the functions of long pAgos is continually advancing, research on short pAgos is still in its early stages.

Computational analyses have revealed that short pAgos are more prevalent in prokaryotes than long pAgos (2,14). Short pAgos only contain MID and PIWI domains, and the PIWI domain is catalytically inactive. However, short pAgos are associated with APAZ (Analog of PAZ) domain-containing proteins in the same operons. The APAZ domains are not homologous to the PAZ domain of long pAgos, but instead to the N domains of long pAgos (15). APAZ domains are frequently fused with nuclease, TIR, or SIR2 domains (1,2,14). It was supposed that nuclease-APAZ proteins compensate for the loss of nuclease activity of short pAgos (3). Recently, it was found that TIR-APAZ and SIR2-APAZ complexed with their associated short pAgos hydrolyze NAD(P)^+^ upon recognition of the target DNA to provide defense against invading DNA, such as phages and plasmids (14,16–18). The nuclease domains fused to APAZ domain include DUF4365 and SMEK domain (2,15). A previous study has shown that SMEK-APAZ and the associated short pAgo form a heterodimeric complex that mediates RNA-guided target DNA cleavage (19). However, the exact function of nuclease-APAZ protein-associated short pAgo systems remains elusive (15).

DUF4365 domain resembles a classic PD-(D/E)XK endonuclease, a fold typically employed to safeguard bacterial genomes against invading DNA by the action of restriction endonucleases (enzymes that cleave DNA into fragments at or near specific recognition sites) (20), and DUF4365-APAZ proteins account for the majority of the nuclease-APAZ proteins (2). In this study, we elucidated the function of a DUF4365-APAZ protein associated with short pAgo system from *Thermocrispum municipal* (named TmuRE-Ago complex). We found that the TmuRE-Ago complex is activated to nonspecifically degrade DNA upon RNA-guided target DNA recognition, suggesting a novel defense strategy of pAgos against invading DNA.

## Materials and methods

### Plasmid construction

The short Ago gene (GenBank accession: WP_028849835.1) and its upstream DUF4365 gene (GenBank accession: WP_028849834.1) from *Thermocrispum municipale* DSM 44069 were synthesized by GenScript (Nanjing, China). For expression of the TmuRE-Ago complex, the DUF4365 gene was inserted into the pET28a vector with an N-terminal 6xHis-tag, followed by another T7 promoter and the short Ago gene. The protein-expression plasmids of TmuRE and TmuRE-Ago complex mutant were constructed by whole-plasmid PCR. All PCR reactions were performed using PrimerSTAR Max DNA Polymerase (Takara).

### Protein expression and purification

Proteins were expressed in the *E. coli* strain BL21(DE3). The protein-expression plasmid of TmuRE-Ago complex was transformed into *E. coli* BL21(DE3) cells. The transformants were inoculated in LB medium supplemented with 50 μg/ml kanamycin and grown for approximately 8 h at 37°C. The cultures were then transferred into fresh LB medium at a ratio of approximately 1:50. When the OD_600_ reached 0.8–1.0, the cultures were induced with 0.4 mM IPTG and shifted to 16°C for 16 h. The cells were harvested and resuspended in lysis buffer (20 mM Tris–HCl, pH 8.0, 500 mM NaCl, 20 mM imidazole, and 1 mM TCEP), lysed by sonication on ice, and centrifuged at 13 500 rpm for 45 min at 4°C. The resulting supernatant was collected, and the protein was purified using Ni-NTA resin (Qiagen) and a gravity column. The resin was washed with lysis buffer supplemented to 50 mM imidazole and eluted with 20 mM Tris–HCl, pH 8.0, 300 mM NaCl, 200 mM imidazole and 1 mM TCEP. The eluted protein was concentrated using a 30K-cutoff concentrator (Millipore) and further purified by size exclusion chromatography on a HiLoad 16/600 Superdex 200 pg column (Cytiva Life Sciences) at room temperature (RT), which was equilibrated in and eluted with SEC buffer (20 mM Tris–HCl, pH 8.0, 300 mM NaCl, and 1 mM TCEP). A total of more than a dozen fractions (1 ml/tube) were collected for the major peak in the chromatography diagram. The four tubes with the highest protein concentrations, corresponding to the middle of the major peak, were selected, combined, and dialyzed twice at 4°C against storage buffer (50 mM Tris–HCl, pH 7.5, 100 mM NaCl, 0.1 mM EDTA, 1 mM DTT, 0.1% TritonX-100 and 50% glycerol) and stored at −20°C. TmuRE and TmuRE-Ago complex mutant were purified using a similar procedure as described above.

To determine the size of the TmuRE Ago complex, the Ni-NTA resin column-purified protein was separated through a Superdex 200 Increase 10/300 GL column (Cytiva Life Sciences) at a flow rate of 0.3 ml/min. Under the same experimental conditions, protein standards including γ-globulin (158 kDa), BSA (66 kDa), ovalbumin (44 kDa), and myoglobin (17 kDa) were also loaded and eluted. A linear calibration curve of standard protein molecular weights against their elution volumes was plotted to determine the molecular weight of the TmuRE Ago complex.

### Thrombin digestion

10 μg of TmuRE-Ago complex or TmuRE was digested by 1 U thrombin (Beyotime, China) overnight at 20°C in the reaction buffer containing 20 mM Tris–HCl, pH 8.0 and 150 mM NaCl. After digestion, the products were analyzed by SDS-PAGE.

### Cleavage assays

Guide nucleic acids and target nucleic acids were synthesized by GenScript (Nanjing, China) and listed in Supplementary Table S1. For determining the ssDNA and dsDNA cleavage activities of the TmuRE-Ago complex under different reaction conditions, M13 single-stranded DNA (ssDNA) and pET28b plasmid were used as substrates in all cleavage assays. M13 ssDNA was purchased from New England Biolabs, while pET28b plasmid was isolated from *E. coli* DH5α using a plasmid purification kit (TIANGEN, China).

In general, 500 nM TmuRE-Ago complex and 500 nM guide nucleic acids (RNA/DNA) were incubated in New England Biolabs (NEB) CutSmart buffer (20 mM Tris-Ac, 50 mM KAc, 10 mM Mg(Ac)_2_, 0.1 mg/ml BSA, pH 7.9) supplemented with 2 mM DTT for 15 min at 37°C. Subsequently, 500 nM complementary target nucleic acids (DNA/RNA) and 100 ng/μl M13 ssDNA or pET28b plasmid were added to the reaction mixture. The reactions (10 μl) were incubated for 30 min and then terminated by adding 2 μl of 6 × TriTrack DNA Loading dye containing 60 mM EDTA (Thermo Fisher Scientific) for agarose gel electrophoresis.

To examine whether TmuRE-Ago complex cleaves the target DNA, we conducted the following experiments. A 5′-FAM-labeled target DNA (FAM-TD-24 nt) were synthesized by GenScript (Nanjing, China). We mixed 500 nM TmuRE-Ago complex with 500 nM guide DNA in NEB CutSmart buffer supplemented with 2 mM DTT, and incubated them at 37°C for 15 min for guide loading. Next, 500 nM 5′-FAM-labeled target DNA and 100 ng/μl additional M13 ssDNA or pET28b plasmid were added, and the reactions (20 μl) were incubated for 30 min. To terminate the reactions, 4 μl of 6 × TriTrack DNA Loading dye was added. Half of the reactions were analyzed by agarose gel electrophoresis to examine the cleavage of additional M13 ssDNA or pET28b plasmid, while the other half was analyzed by 10% denaturing polyacrylamide gel containing 7 M urea to examine the cleavage of 5′-FAM-labeled target DNA, and visualized using a fluorescence imaging system (CLINX, China).

To examine the cleavage of additional short ssDNA and dsDNA by TmuRE-Ago, we conducted the following experiments. We incubated 500 nM TmuRE-Ago complex and 500 nM guide RNA in NEB CutSmart buffer supplemented with 2 mM DTT for 15 min at 37°C, and then added 500 nM target DNA and additional short ssDNA (ssDNA-1 or ssDNA-2) or dsDNA (dsDNA-1 or dsDNA-2) into the reaction mixtures. The reactions (10 μl) were incubated for 30 min, and then terminated by adding 2 μl of 6 × TriTrack DNA Loading dye before being examined by 10% polyacrylamide gel.

### Electrophoretic mobility shit assay (EMSA)

To examine the guide binding ability of TmuRE-Ago complex, 50 nM of three different 3′-FAM labeled nucleic acid substrates (5′-U Guide RNA, 5′-A Guide RNA, and 5′-T Guide DNA) were incubated with a gradient of TmuRE-Ago complex (50, 100, 200, 400, 800 nM), respectively, in 10 μl mixture containing 20 mM Tris-Ac pH 7.9, 50 mM KAc, 10 mM Mg(Ac)_2_, and 2 mM DTT at 37°C for 20 min. After incubation, the reaction samples were mixed with 2 μl of 6 × TriTrack DNA Loading dye supplemented with 60 mM Mg(Ac)_2_. The reaction mixtures were analyzed by 8% polyacrylamide gel in TAE buffer (40 mM Tris-acetate, 1 mM EDTA) supplemented with 5 mM Mg(Ac)_2_.

To analyze the target binding specificity of TmuRE-Ago complex, 480 nM TmuRE-Ago complex was pre-incubated with 200 nM of unlabeled 5′-U guide RNA at 37°C for 15 min. Subsequently, aliquots of the mixture were diluted to final concentrations of 60, 120 and 240 nM TmuRE-Ago complex, respectively. These diluted mixtures were then incubated with 50 nM of 5′-FAM labeled target DNA (FAM-TD), non-target DNA (FAM-NTD), and target RNA (FAM-TR) at 37°C for 15 min, respectively. Alternatively, 120 nM of TmuRE-Ago complex was pre-incubated with 50 nM of 3′-FAM-labeled guide RNA (GR-FAM) at 37°C for 15 min. The resulting mixtures were then incubated with a gradient of target DNA (TD), non-target DNA (NTD), and target RNA (TR) at concentrations of 25, 50, 100 and 200 nM at 37°C for 15 min. The reaction mixtures were analyzed using 8% polyacrylamide gel in TAE buffer (40 mM Tris-acetate, 1 mM EDTA) supplemented with 5 mM Mg(Ac)_2_ and 1 mM DTT.

### dsDNA reporter cleavage assays

To perform the dsDNA reporter cleavage assays, 100 nM TmuRE-Ago complex and 100 nM guide RNA were incubated in a reaction buffer containing 20 mM HEPES (pH 7.5), 100 mM KAc, 5 mM Mg(Ac)_2_, and 2 mM DTT for 15 min at 37°C. Then, the indicated amount of target DNA and 500 nM dsDNA reporter were added to the reaction mixtures, unless otherwise specified. The reactions (10 μl) were incubated for 60 min and then terminated by adding 1 μl of 100 mM EDTA. The fluorescence signals were visualized and captured using a fluorescence imaging system (CLINX, China). Next, the reactions were transferred to a 384-well microplate, and the fluorescence was measured using a FlexStation 3 Multi-Mode Microplate Reader (excitation: 485 nm, emission: 525 nm).

## Results

### Diverse short pAgos associated with APAZ domain-containing effector proteins

Previous bioinformatics analyses have shown that short pAgos account for approximately 60% of the total pAgos (2). Typically, the short pAgos are associated with an APAZ domain-containing protein. Recently, the functions of two types of APAZ domain-containing proteins (TIR-APAZ and SIR2-APAZ) have been identified (14,16). Both proteins interact with their associated short pAgos to form a heterodimeric complex, which hydrolyzes NAD(P)^+^ to induce cell death after the associated short pAgos recognize and bind to the target DNA (14,16). The APAZ domain-containing proteins appear to function as the effector proteins in the short pAgo systems. In this study, we summarize diverse APAZ domain-containing protein-associated short pAgo systems based on previous bioinformatics analyses (1,2). Based on the different effector domains of the APAZ domain-containing proteins, we divided the short pAgo systems into three groups: nuclease effector-associated short pAgos, NAD(P)ase effector-associated short pAgos, and double-effector-associated short pAgos (Figure 1B). The nuclease effectors consist of APAZ domain fused to various nuclease domains including DUF4365, HNH, SMEK, and REase. NAD(P)ase effectors consist of APAZ domain fused to TIR or SIR2 domain, and they are sometimes fused to the associated short pAgo to form a single protein. Additionally, two APAZ domain-containing proteins contain two different effector domains. One contains a SIR2 domain and a histidine kinase domain, while the other contains a REase domain and a TIR domain. The APAZ domain-containing proteins with two effector domains may perform two different functions simultaneously after activation.

### TmuRE-Ago complex nonspecifically degrades DNA upon RNA-guided target DNA recognition

**Figure 2.**
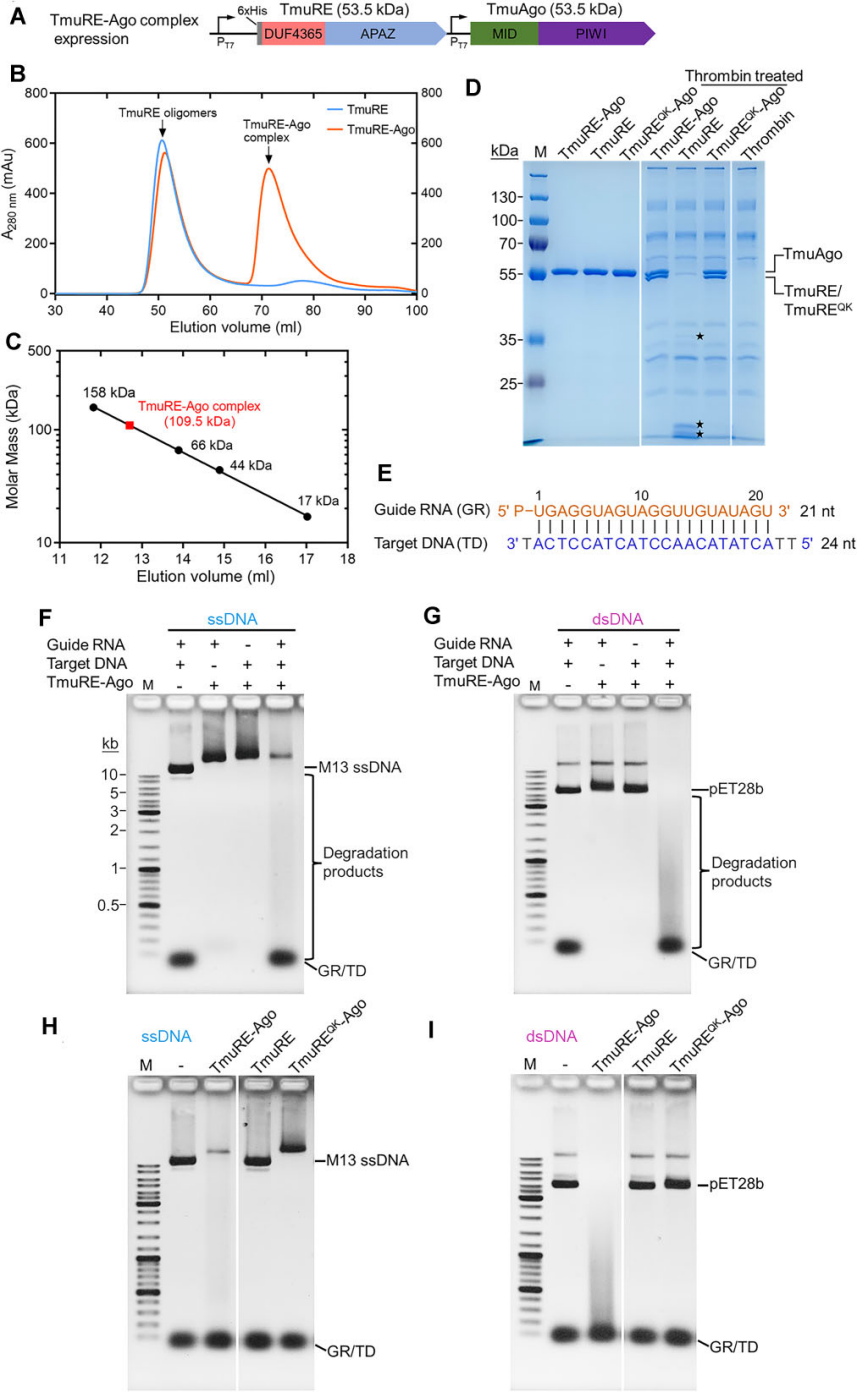
TmuRE-Ago complex exhibits non-specific DNA cleavage activity upon RNA-guided target DNA recognition. (**A**) Schematic diagram of the plasmid constructed for TmuRE-Ago complex expression. (**B**) TmuRE-Ago complex and TmuRE were purified and analyzed by size exclusion chromatography on a HiLoad 16/600 Superdex 200 pg column. (**C**) Determination of the molecular mass of TmuRE-Ago complex using the elution volumes of four protein standards: γ-globulin (158 kDa), BSA (66 kDa), ovalbumin (44 kDa) and myoglobin (17 kDa) separated through a Superdex 200 Increase 10/300 GL column. (**D**) SDS-PAGE analysis of TmuRE-Ago complex, TmuRE and mutant TmuRE^QK^-Ago complex (mutations Q62A/K64A in TmuRE) before or after thrombin treatment. Black stars indicate TmuRE fragments generated by thrombin cleavage. (**E**) Schematic representation of the guide RNA and target DNA used in (F–I). (**F, G**) TmuRE-Ago complex is activated to non-specifically cleave ssDNA (**F**) and dsDNA (**G**) in the presence of both guide RNA and target DNA. Data are representative of two independent experiments. (**H, I**) Both TmuRE and TmuAgo are required for the cleavage activities of ssDNA (**H**) and dsDNA (**I**). In the assays, the amounts of TmuRE-Ago complex, TmuRE and mutant TmuRE^QK^-Ago complex were all at 500 nM. GR/TD: guide RNA/target DNA hybrids. Data are representative of two independent experiments.

To obtain the TmuRE-Ago complex, both the DUF4365-APAZ (TmuRE) and Ago (TmuAgo) from *Thermocrispum municipal* were co-expressed in *E. coli* strain BL21(DE3) under the control of the T7 promoter, and a 6 × His tag was added at the N-terminus of TmuRE to enable the pull-down of untagged TmuAgo (Figure 2A). The His-tagged TmuRE and TmuAgo were co-purified using a Ni^2+^-affinity column, followed by size exclusion chromatography on a Superdex 200 column (Figure 2B). The sequence alignment indicates that Q62 and K64 are potential active site residues in the DUF4365 domain of TmuRE (Supplementary Figure S1). We also expressed and purified the TmuRE and TmuRE^QK^-Ago complex with an inactive TmuRE mutant (Q62A/K64A) using a similar procedure. We observed that TmuRE was prone to aggregate at room temperature, thus the protein was eluted at about 50 ml (Figure 2B), which facilitate the TmuRE-Ago complex purification. To determine the molecular weight of TmuRE-Ago complex, the complex was subject to gel filtration analysis with a Superdex 200 Increase 10/300 GL column (Supplementary Figure S2). Based on the elution volumes of the four protein standards, the calculated molecular weight of TmuRE-Ago complex was 109.5 kD (Figure 2C). We next analyzed the TmuRE-Ago complex, TmuRE, and TmuRE^QK^-Ago complex by SDS-PAGE. His-tagged TmuRE had a similar molecular weight to TmuAgo. To distinguish the two proteins, the His tag was removed by thrombin, and two bands were observed from the complexes of TmuRE-Ago and TmuRE^QK^-Ago (Figure 2D), further demonstrating that TmuRE and TmuAgo form a heterodimeric complex. This result is also consistent with previous studies indicating that short Agos and their associated APAZ proteins form heterodimeric complexes with a 1:1 stoichiometry (14,16,19).

In recent years, a large number of novel anti-phage defense systems have been identified (21–25), most of which provide immunity through abortive infection (26). These defense systems utilize nuclease effector proteins to degrade genome DNA, or TIR and SIR2 effector proteins to deplete NAD^+^, serving as crucial mechanisms to trigger cell death. Short pAgo systems associated with TIR-APAZ and SIR2-APAZ proteins have been identified to induce abortive infection by depleting NAD^+^ upon detection of invading DNA (14,16). Although Nuclease-APAZ proteins were previously thought to compensate for the lack of nuclease activity of the associated short pAgos and specifically cleave the target DNA (14,19), we speculated that nuclease-APAZ proteins associated with pAgo systems might be activated to induce abortive infection by nonspecifically cleaving DNA. Following previous studies (14,27), we initially tested whether the short pAgo systems could be preloaded with nucleic acid guide, and then activated by complementary nucleic acid target. A 5′-phosphorylated (5′-P) 21-nt guide RNA which has been used for studying another short pAgo system (14), was tested first. The TmuRE-Ago complex was incubated with the 21-nt guide RNA, followed by incubation with a 24-nt target DNA and additional M13 ssDNA or pET28b double-stranded DNA (dsDNA) plasmid. The cleavage of M13 ssDNA and the plasmid DNA were detected by agarose gel electrophoresis (Figure 2E–G). Notably, although the guide RNA has no sequence match to the M13 ssDNA and pET28b plasmid, TmuRE-Ago complex can efficiently degrade both the M13 ssDNA and plasmid dsDNA in the presence of guide RNA and target DNA (Figure 2F-G and Supplementary Figure S3). The cleavage by the TmuRE-Ago complex depends on both guide RNA and target DNA, as no cleavage activity was observed in the absence of guide RNA or target DNA (Figure 2F-G). These results suggest that RNA-guided target DNA recognition activates the TmuRE-Ago complex for nonspecific DNA degradation. Compared to the cleavage activity of TmuRE-Ago complex, the TmuRE and TmuRE^QK^-Ago complex could not cleave M13 ssDNA and plasmid dsDNA in the presence of guide RNA and target DNA (Figure 2H-I), indicating that both the TmuRE and TmuAgo subunits are required for DNA degradation. In addition, as migration shift of M13 ssDNA gel bands was observed in Figure 2F and H, we examined the binding of TmuRE-Ago complex to M13 ssDNA or pET28b plasmid in the absence of guide RNA and target DNA (Supplementary Figure S4). The results showed a strong binding of the TmuRE-Ago complex to M13 ssDNA in the absence of guide RNA and target DNA (Supplementary Figure S4). However, the TmuRE-Ago complex remains inactive for cleavage in this state (Figure 2F-G).

We also designed experiments to evaluate whether the TmuRE-Ago complex could cleave the target DNA in a manner similar to long pAgos (Supplementary Figure S5A-C). We observed no cleavage of the target DNA in the presence of additional ssDNA (Supplementary Figure S5C). However, in the absence of additional DNA, the TmuRE-Ago complex exhibited slight cleavage activity on the target DNA (Supplementary Figure S5C). Compared with additional DNA, the target DNA is not significantly cleaved by TmuRE-Ago complex in our assays (Supplementary Figure S5B-C), unlike the specific cleavage of the target DNA by long pAgos (4,5).

### The preference of the TmuRE-Ago complex for the guide and target nucleic acids

**Figure 3.**
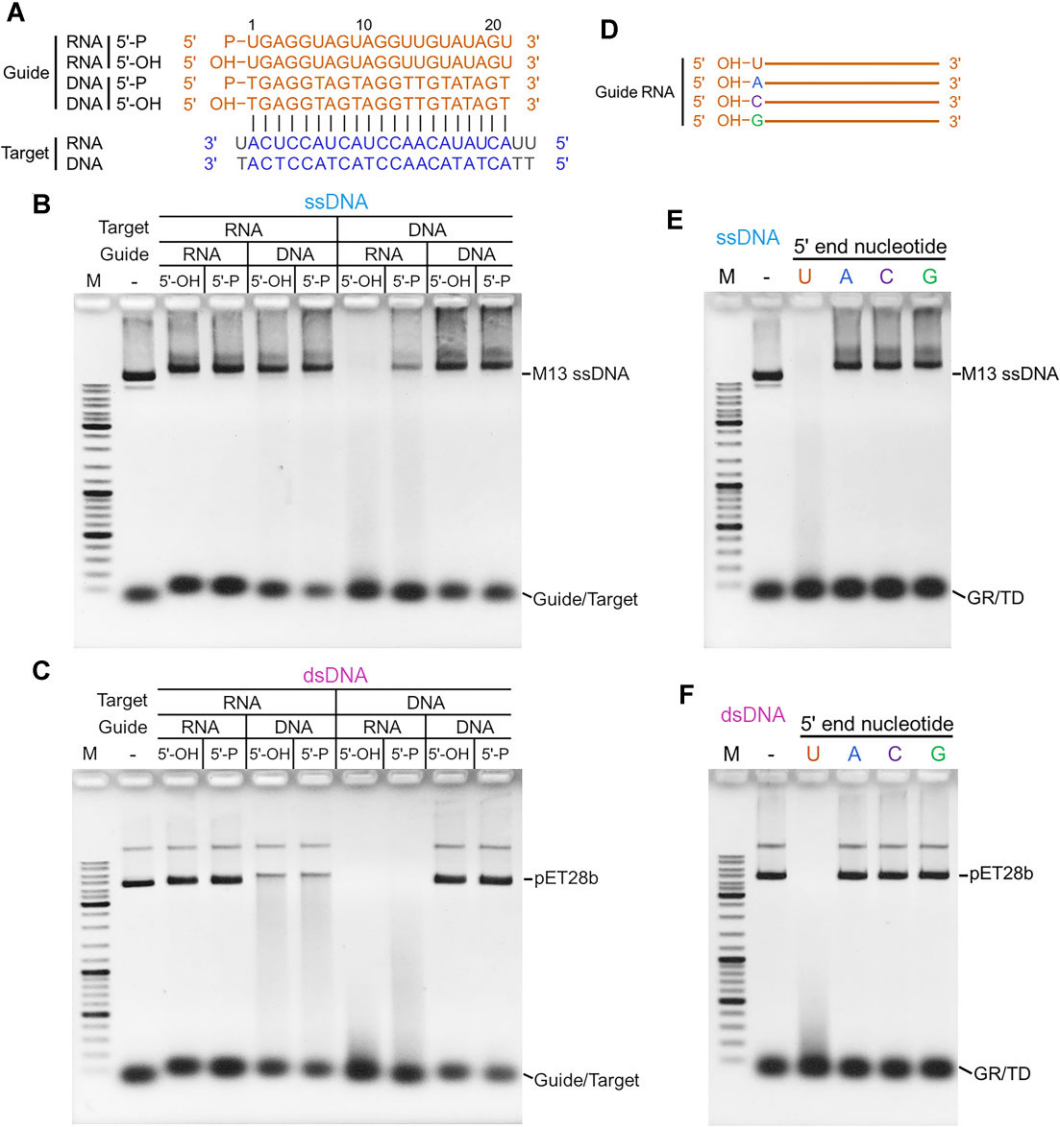
Preferences of the TmuRE-Ago complex toward guide and target nucleic acids. (**A**) Schematic representation of the guide and target nucleic acids used in (B, C). (**B, C**) The TmuRE-Ago complex prefers RNA guides and DNA targets to efficiently cleave ssDNA (**B**) and dsDNA (**C**). Data are representative of two independent experiments. (**D**) Schematic representation of the guide RNAs with different 5′ end nucleotides used in (E, F). The target DNAs used were correspondingly changed to guarantee complementary base pairing to guide RNAs. (**E, F**) The TmuRE-Ago complex exhibits efficient cleavage activities towards ssDNA (**E**) and dsDNA (**F**), only when the guide RNA with a uridine nucleotide at its 5′ end was used. GR/TD: guide RNA/target DNA hybrids. Data are representative of two independent experiments.

We demonstrated that the activation of the TmuRE-Ago complex depended on RNA-guided target binding. We subsequently investigated the complex's preferences for guide and target nucleic acids (Figure 3A). The results revealed that M13 ssDNA and plasmid dsDNA were efficiently degraded in the presence of RNA guides and DNA targets (Figure 3A–C), indicating that the TmuRE-Ago complex prefers RNA guides and DNA targets. Furthermore, the TmuRE-Ago complex displayed higher cleavage activity with 5′-OH RNA guides than 5′-P RNA guides (Figure 3A–C). It is noteworthy that the TmuRE-Ago complex also exhibits weak cleavage activity in the presence of DNA guide and RNA target (Figure 3C). Given that both the guides and the targets tested were similar in length, it is possible that the TmuRE-Ago complex might not efficiently distinguish the guides from the targets in these assays.

Next, we investigated the complex's preferences for the nucleotide at the 5′ end of the guide RNA. Interestingly, we found that M13 ssDNA and plasmid DNA can only be cleaved when the 5′ end of the guide RNA is a uridine nucleotide (Figure 3D–F), indicating that the TmuRE-Ago complex exhibits stringent 5′-nucleotide recognition for guide RNA.

We conducted further analysis of the guide loading properties of TmuRE-Ago complex through EMSA experiments. The results showed that TmuRE-Ago complex exhibits a stronger affinity to 5′-U Guide RNA compared to 5′-A Guide RNA and 5′-T Guide DNA (Supplementary Figure S6A-B), consistent with the cleavage activity results of TmuRE-Ago complex. However, it's worth noting that the TmuRE-Ago complex can also bind to 5′-A Guide RNA and 5′-T Guide DNA, but cannot be effectively activated in the presence of these two guides (Figure 3C and F). These results suggest additional regulations between guide binding and cleavage activation of the TmuRE-Ago complex.

We also evaluated the target binding of TmuRE-Ago complex pre-loaded with guide RNA and obtained interesting results. Upon target DNA recognition, the binding of TmuRE-Ago complex to guide RNA is weakened, or the binding mode to the RNA/DNA duplex by TmuRE-Ago complex is altered, making it difficult to detect the binding using EMSA (Supplementary Figure S7A-B). Non-target DNA showed little effect on the binding of TmuRE-Ago complex to guide RNA, while target RNA also released the guide RNA from TmuRE-Ago complex as indicated by the EMSA results (Supplementary Figure S7A-B). Similarly, weak target binding by short pAgo complex was also observed in the recent work (28).

### TmuRE-Ago complex exhibits endonuclease but not exonuclease activity

TmuRE-Ago complex cleaves both circular ssDNA (M13 ssDNA) and dsDNA (plasmid pET28b), demonstrating its endonuclease activity. However, its potential exonuclease activity remains unclear, as it cleaves DNA into very small fragments (Figure 3C). To investigate this, we compared the cleavage activities of TmuRE-Ago complex, T4 DNA polymerase (a 3′-5′ exonuclease), and T5 exonuclease (a 5′-3′ exonuclease) by digesting a 500 bp linear DNA fragment. T4 DNA polymerase and T5 exonuclease cannot cleave circular plasmid DNA but can efficiently cleave the linear DNA fragment (Supplementary Figure S8A-B). Interestingly, the intermediate products (ssDNAs) migrated upwards in the gel, disappearing over time (Supplementary Figure S8B). In contrast, the intermediate products generated by TmuRE-Ago complex did not migrate upwards (Supplementary Figure S8C-D), indicating the absence of ssDNA intermediate products. Even with extended reaction times or increased enzyme concentrations, the intermediate products were not completely degraded (Supplementary Figure S8C-D). These results suggested that TmuRE-Ago complex lacks exonuclease activity.

### TmuRE-Ago complex is sensitive to mismatches between guide and target nucleic acids

**Figure 4.**
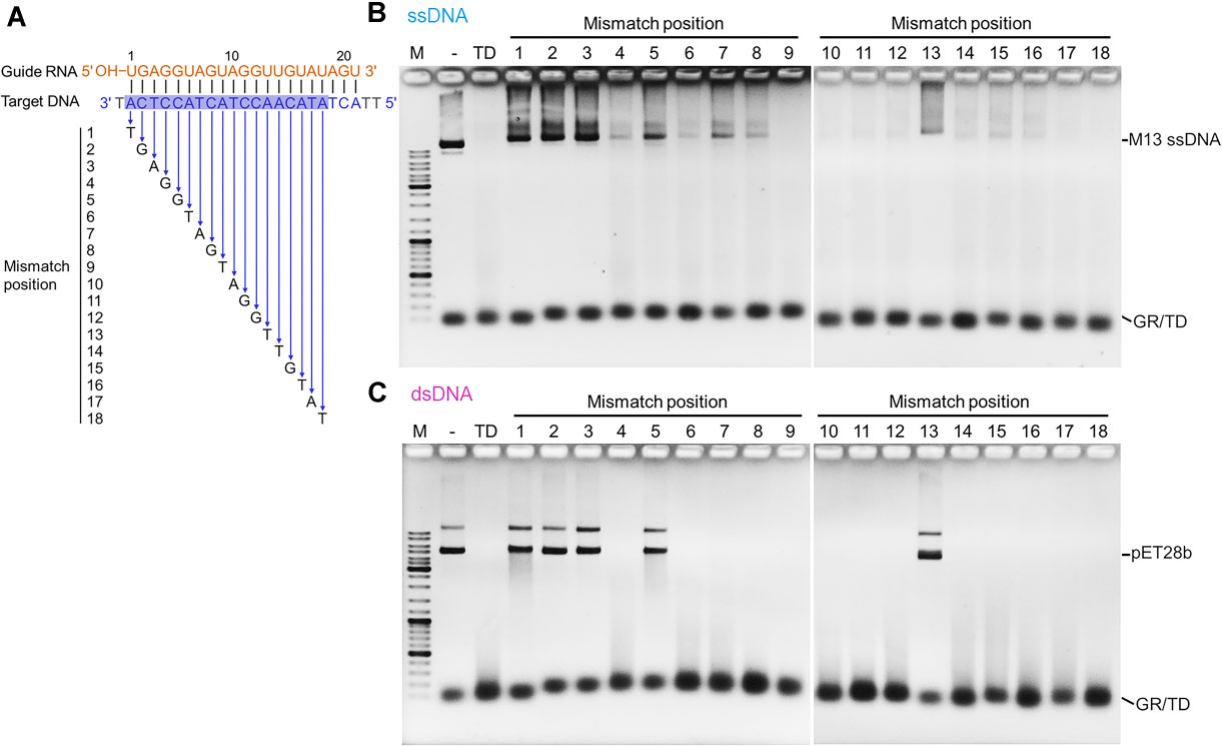
Effects of single-nucleotide mismatches between guide RNA and target DNA on cleavage activity of the TmuRE-Ago complex. (**A**) Schematic representation of the guide RNA and target DNAs used in (B, C). The guide sequences used were the same while the target sequences were varied as shown in the figure to form mismatches. (**B, C**) Single-nucleotide mismatches at different positions exhibit different effects on cleavage activities towards ssDNA (**B**) and dsDNA (**C**). Single-nucleotide mismatches were introduced at positions 1–18 counting from the 5′ end of the guide RNA, and their effect on cleavage efficiency was tested. GR/TD: guide RNA/target DNA hybrids. Data are representative of two independent experiments.

Previous studies have shown that mismatches can reduce the cleavage efficiency of pAgos to some extent (8,14,29). In this study, we also investigated the effects of mismatches on the cleavage activity of TmuRE-Ago complex. The results showed that a single-nucleotide mismatch at positions 1–3, 5 or 13, as well as most two consecutive nucleotide mismatches at the front- and middle-region of the guide/target, abolished the ssDNA and dsDNA cleavage activity of the TmuRE-Ago complex (Figure 4 and Supplementary Figure S9), suggesting that TmuRE-Ago complex is sensitive to mismatches. Recent structural studies of the TIR-APAZ protein-associated short pAgos in complex with guide/target nucleic acids revealed that many residues of the protein complexes interact with the similar regions, and interestingly, two residues interact with position 13 (28,30). However, single mismatches at these positions do not dramatically affect the activity of the TIR-APAZ associated short pAgo complexes (14).

### The cleavage activity of TmuRE-Ago complex is affected by the overhang sequences of target DNA

**Figure 5.**
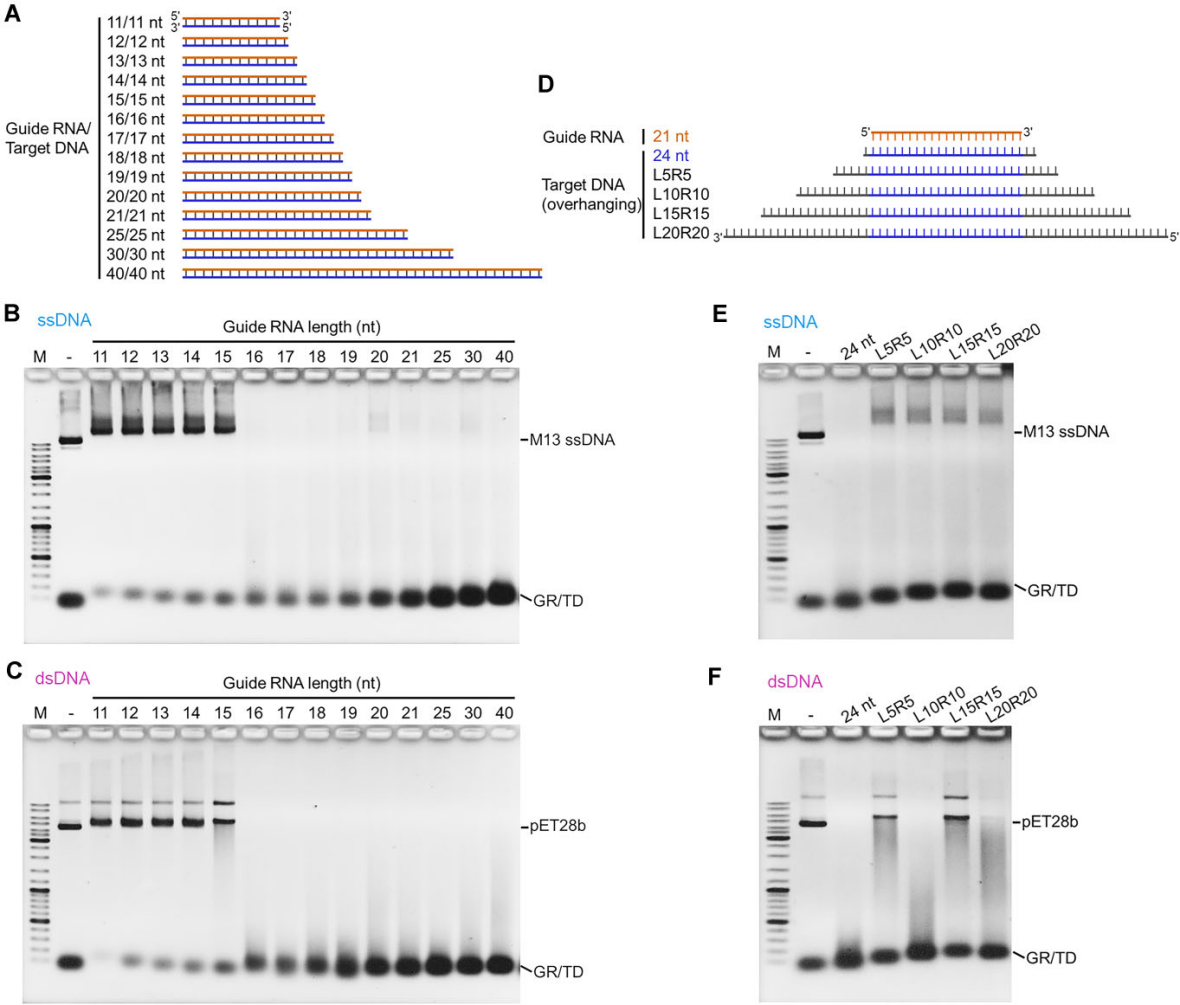
Effects of the lengths of guide RNA and target DNA on cleavage activity of the TmuRE-Ago complex. (**A**) Schematic representation of the guide RNAs and target DNAs used in (B, C). Guide RNAs with different lengths and corresponding target DNAs with the same lengths are used in each assay. (**B, C**) Effects of guide RNA lengths on ssDNA (**B**) and dsDNA (**C**) cleavage activity. Data are representative of two independent experiments. (**D**) Schematic representation of the guide RNA and target DNA with overhang sequences of different lengths used in (E, F). The length of the guide RNA used was fixed at 21 nt, while the target DNAs with overhang sequences of different lengths were used in each experiment. (**E, F**) Effects of the overhang sequences of the target DNAs on ssDNA (**E**) and dsDNA (**F**) cleavage activity. GR/TD: guide RNA/target DNA hybrids. Data are representative of two independent experiments.

Previous studies have shown that the length of guide nucleic acids can influence the cleavage activity of pAgos (7,8,29,31,32). In this study, we designed a set of guide RNA and fully complementary target DNA with varying lengths (Figure 5A) to investigate their effects on the cleavage activity of TmuRE-Ago complex. Results showed that TmuRE-Ago complex was only activated by guide RNA/target DNA pair with a length of at least 16 bp (Figure 5B-C). We further examined the cleavage activity of TmuRE-Ago complex using the 21-nt guide RNA and target DNAs of various lengths. We designed a series of target DNAs with overhang sequences of varying lengths at both the 3′ and 5′ sides (Figure 5D). The results showed that TmuRE-Ago complex only exhibited slight ssDNA cleavage activity in the presence of target DNAs with overhang sequences on both sides (Figure 5E). However, TmuRE-Ago complex showed varying dsDNA cleavage activity in the presence of target overhangs with various lengths (Figure 5F). These results indicated that the overhang sequences in the target DNA can significantly affect the cleavage activity of the TmuRE-Ago complex, while the underlying mechanism remains to be explored. In addition to the lengths of the target overhangs, the sequences and potential secondary structures of these overhangs may also have impacts on the TmuRE-Ago complex activity.

### The potential of TmuRE-Ago complex used for nucleic acid detection

**Figure 6.**
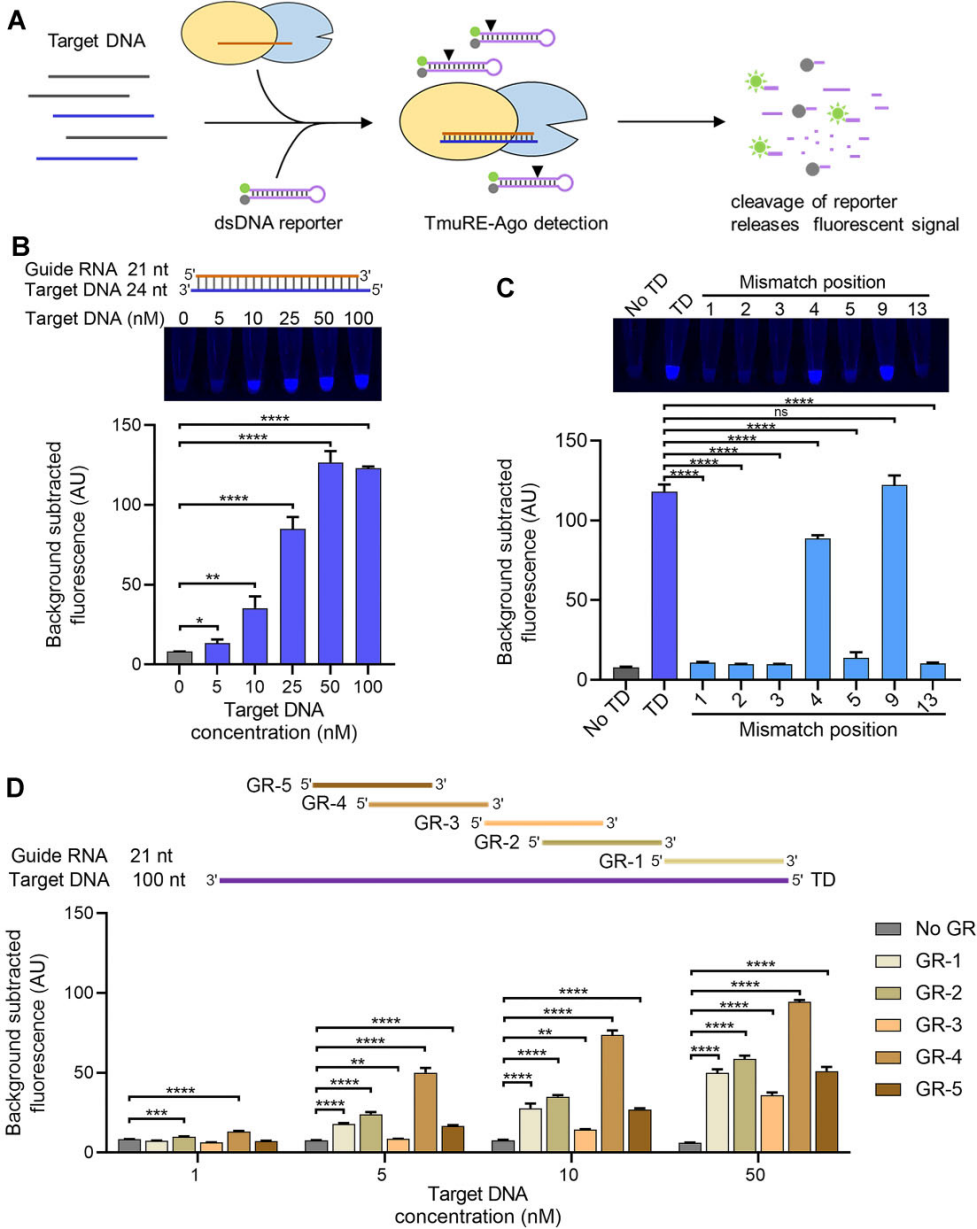
Nucleic acid detection by the TmuRE-Ago complex. (**A**) Schematic illustration of the principle of nucleic acid detection using the TmuRE-Ago complex. RNA-guided TmuRE-Ago complex is activated to cleave designed dsDNA reporter upon target detection, releasing a fluorescent signal. (**B**) Fluorescence detection of different concentrations of 24-nt target DNA by the TmuRE-Ago complex. (**C**) The sensitivity of the TmuRE-Ago complex-based nucleic acid detection to single-nucleotide mismatches. (**D**) Fluorescence detection of 100-nt target DNA at varying concentrations using the TmuRE-Ago complex with guide RNAs targeting different regions of the target DNA. GR: guide RNA; TD: target DNA. The data are shown as mean ± SD from three technical replicates. Two-tailed P values were calculated using unpaired *t*-tests: ns, not significant, ∗*P*< 0.05, ∗∗*P*< 0.01, ∗∗∗*P*< 0.001 and ∗∗∗∗*P*< 0.0001.

As the TmuRE-Ago complex can be activated to non-specifically cleave DNA upon RNA-guided target DNA recognition, we wondered whether it could be used as a nucleic acid detection tool similar to CRISPR-Cas12 and -Cas13 (33,34). First, we examined whether the TmuRE-Ago complex could efficiently cleave additional short ssDNA or dsDNA (Supplementary Figure S10A-B). The results showed that it can efficiently cleave short dsDNAs but not ssDNAs (Supplementary Figure S10). Based on these results, we designed a fluorescence reporter DNA based on the short dsDNAs. The principle of the TmuRE-Ago complex-based nucleic acid detection is shown in Figure 6A. When the RNA-guided TmuRE-Ago complex recognizes and binds to the target DNA, it will be activated and non-specifically cleave the reporter DNA to release the fluorescence signal. We first used 21-nt guide RNA and 24-nt target DNA to verify the feasibility of the method. The results showed that the TmuRE-Ago complex can detect as low as 5 nM target DNA (Figure 6B), which is comparable to the sensitivity of CRISPR-based tools and the TIR-APAZ-associated short Ago system-based method without a pre-amplification step (35). Next, we evaluated the sensitivity to single-nucleotide mismatches. Interestingly, TmuRE-Ago complex-based nucleic acid detection is highly sensitive to single mismatches at certain positions (Figure 6C). The results detected by fluorescence assays are also consistent with the agarose gel results shown in Figure 4. To further verify the feasibility of TmuRE-Ago complex used in nucleic acid detection, we used a 100-nt ssDNA as the target DNA. We found that the detection efficiency by TmuRE-Ago complex varied when paired with guide RNAs targeting distinct regions of the target DNA, with a detection limit of 1 nM (Figure 6D and Supplementary Figure S11). These results further demonstrate the potential of the TmuRE-Ago complex for nucleic acid detection. One limitation is that TmuRE-Ago complex only targets ssDNA and cannot be used for dsDNA detection directly (Supplementary Figure S12). Techniques such as using exonucleases to convert amplified dsDNA into ssDNA or using DNA polymerases with strand displacement activity to amplify DNA are possible solutions to obtain ssDNA targets (14,36,37).

## Discussion

Short pAgo systems make up the majority of the pAgo family, and nuclease-associated pAgo systems are an important subclass of short pAgos. However, their functions and regulation have largely remained unknown. Previous studies have characterized many long pAgos that specifically cleave target ssDNA or RNA through guide ssDNA- or RNA-mediated recognition. In contrast, short pAgos lack cleavage activity, and their associated nuclease proteins were considered to compensate for their activities. Indeed, a SMEK nuclease-associated short pAgo system was tested for its ability to cleave target nucleic acids (19). However, the exact function of the system remained unknown. In this study, we elucidated the biological function of a DUF4365 nuclease-associated short pAgo system, showing that it is activated to nonspecifically degrade DNA through guide RNA-mediated target DNA recognition. Our biochemical results also demonstrated that a uridine nucleotide at the 5′ end of the guide RNA is necessary for the cleavage activity of the TmuRE-Ago complex; and single mismatches at several positions can completely abolish the cleavage activity cleavage. Taken together, the TmuRE-Ago complex has stringent requirements for guide RNA and target DNA.

Recently, two types of short pAgo systems containing TIR or SIR2-APAZ proteins have been characterized (14,27). Both systems provide immunity by inducing abortive infection through NAD^+^ depletion upon detection of invading DNA. In addition, a pseudo-short pAgo system, containing two associated proteins, in which one is the membrane effector, provides antiviral defense by inducing abortive infection through membrane depolarization (15,38). In this study, we demonstrated that the DUF4365-APAZ protein-associated short pAgo system is activated to nonspecifically degrade DNA upon target recognition. Such nonspecific DNA cleavage may also provide anti-phage defense through abortive infection.

pAgo proteins and CRISPR-Cas systems are two types of programmable nucleases that can be engineered for target recognition and cleavage. Besides gene editing, Cas12 and Cas13 have demonstrated wide applications in nucleic acid detection due to their collateral cleavage activities (33,34). Short pAgo systems possess effector proteins that are activated upon target recognition, making them suitable for nucleic acid detection. Indeed, TIR-APAZ protein-associated short pAgo systems have been engineered for this purpose by hydrolyzing etheno-NAD (ϵ-NAD), achieving a sensitivity comparable to that of CRISPR-based tools. In this study, we demonstrated that the DUF4365-APAZ protein-associated short pAgo system exhibits robust nonspecific cleavage activity upon target recognition. Based on our results, we established a new detection tool that is similar to CRISPR-based tools, cleaving nucleic acid reporters to release fluorescent signals. Our findings might also expand the applications of short pAgo systems in nucleic acid detection. Since the TmuRE-Ago complex is sensitive to single-nucleotide mismatches, it has potential to be used in single-nucleotide polymorphism (SNP) detection, similar to CRISPR-Cas14-based method (36). In addition, Since TmuRE-Ago complex only recognizes ssDNA targets, it can be coupled with DNA polymerases that have strand displacement activity to detect target nucleic acid. For example, a method is similar to Cas12a coupled with phi29 DNA polymerase to detect microRNAs (37).

## Supplementary Material

gkad1145_supplemental_file

## Data Availability

The data underlying this article are available in the article and in its online supplementary material.
