# Supplementary material for: The nuclease-associated short prokaryotic Argonaute system nonspecifically degrades DNA upon activation by target recognition
Source: Nucleic Acids Res. 2023 Dec 4;52(2):844–55. doi: 10.1093/nar/gkad1145 (PMC10810196; doi:10.1093/nar/gkad1145)
Supplement: gkad1145_supplemental_file [file gkad1145_supplemental_file.pdf]

Supplementary Material

for

The nuclease-associated short prokaryotic Argonaute system nonspecifically degrades DNA upon activation by target recognition

Xueling Lu[1], Jun Xiao[2], Longfei Wang[2], Bin Zhu[1,3]*, Fengtao Huang[1]*

[1]Key Laboratory of Molecular Biophysics, the Ministry of Education, College of Life Science and Technology, Huazhong University of Science and Technology, Wuhan, Hubei 430074, China;

[2]School of Pharmaceutical Sciences, Wuhan University, Wuhan, China

[3]Shenzhen Huazhong University of Science and Technology Research Institute, Shenzhen 518063, China

*To whom correspondence should be addressed. Email: bin_zhu@hust.edu.cn; huang_fengtao@126.com

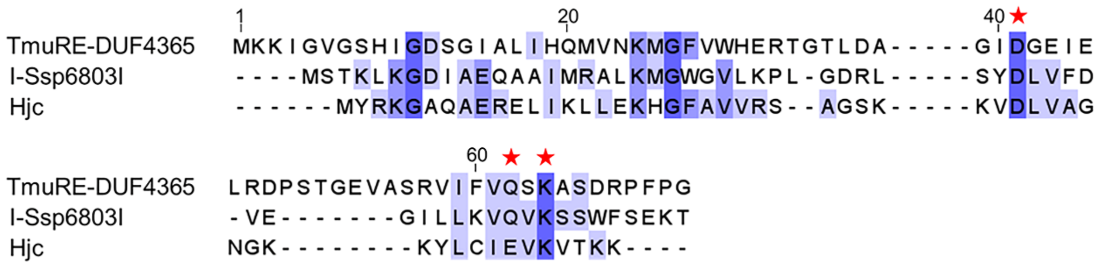

Figure S1. Sequence alignment of the DUF4365 domain of TmuRE with other PD-(D/E)XK endonucleases, including I-Ssp6803I (1) and Hjc (2). HHpred (3) analysis reveals homology between the DUF4365 domain of TmuRE and PD-(D/E)XK endonucleases I-Ssp6803I and Hjc. The predicted active site residues are indicated with red stars.

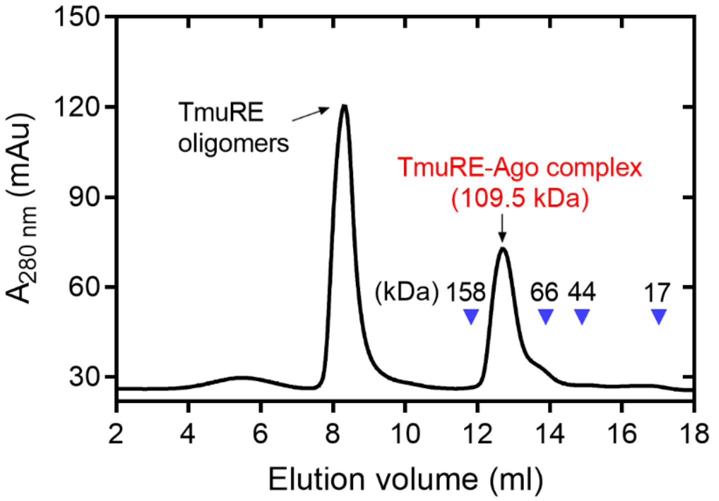

Figure S2. Elution profile of the purified TmuRE-Ago complex determined by gel filtration on a Superdex 200 Increase 10/300 GL column, with the elution volumes of standard proteins of known molecular weight indicated.

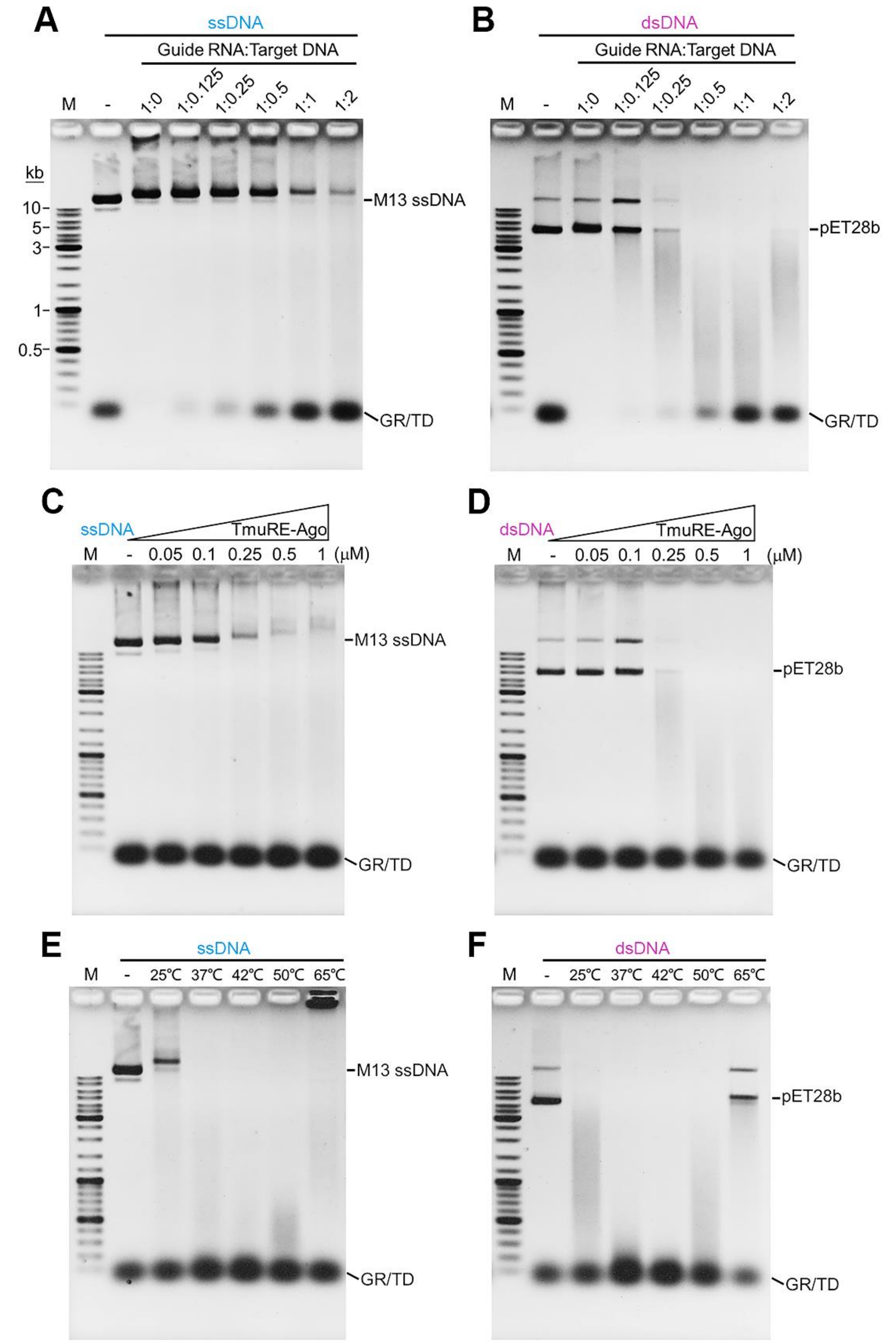

Figure S3. Investigation of cleavage activity of TmuRE-Ago complex under different reaction conditions. (A-B) Effects of different molar ratios of guide RNA: target DNA on ssDNA (A) and dsDNA (B) cleavage activity. (C-D) ssDNA (C) and dsDNA (D) were cleaved by different concentrations of TmuRE-Ago complex. (E-F) Effects of the reaction temperatures on ssDNA (E) and dsDNA (F) cleavage activity. Data are representative of two independent experiments.

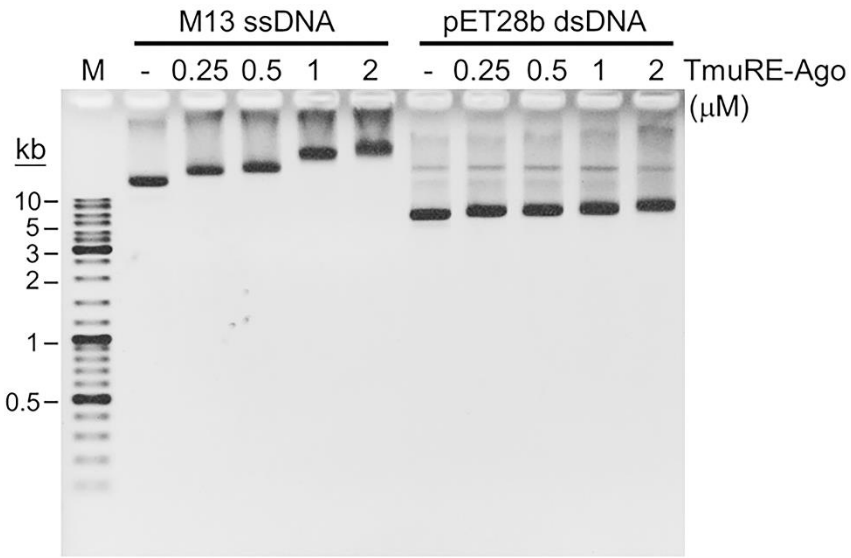

Figure S4. Binding of TmuRE-Ago complex to M13 ssDNA or pET28b plasmid. 100 ng of M13 ssDNA or pET28b plasmid was incubated with indicated concentrations of TmuRE-Ago complex in a 10 μl mixture containing 20 mM Tris-Ac pH 7.9, 50 mM KAc, 10 mM $Mg(Ac)_2$, and 2 mM DTT at 37°C for 15 min. The reaction samples were analyzed by 1% agarose gel.

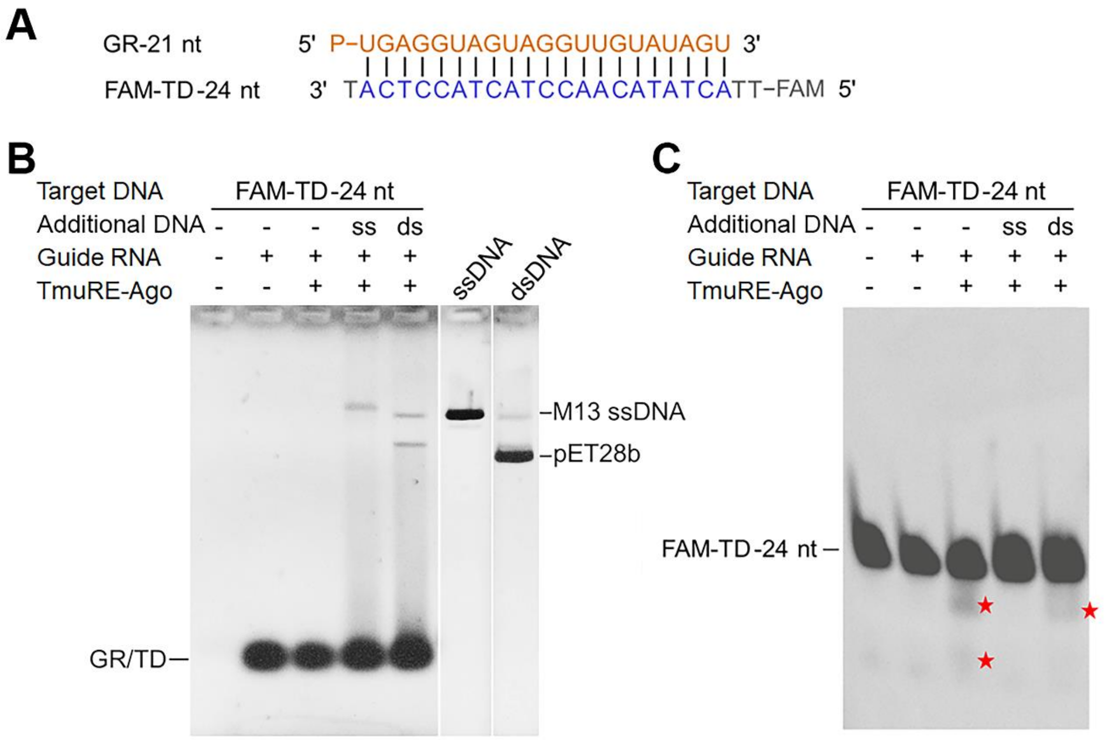

Figure S5. Examination of the cleavage of target DNA and additional DNA by TmuRE-Ago complex. (A) Schematic representation of the guide RNA and 5'-FAM labeled target DNA used in the experiment. (B) Native agarose gel analysis of the cleavage of additional DNA by TmuRE-Ago complex. (C) Denaturing polyacrylamide gel analysis of the cleavage of 5'-FAM-labeled target DNA. Reaction mixtures (20 µl) containing 500 nM TmuRE-Ago, 500 nM guide RNA, and 500 nM target DNA were incubated with or without 100 ng/µl M13 ssDNA or pET28b plasmid at 37°C for 30 min. Red stars indicate cleavage products. GR/TD: guide RNA/target DNA hybrids.

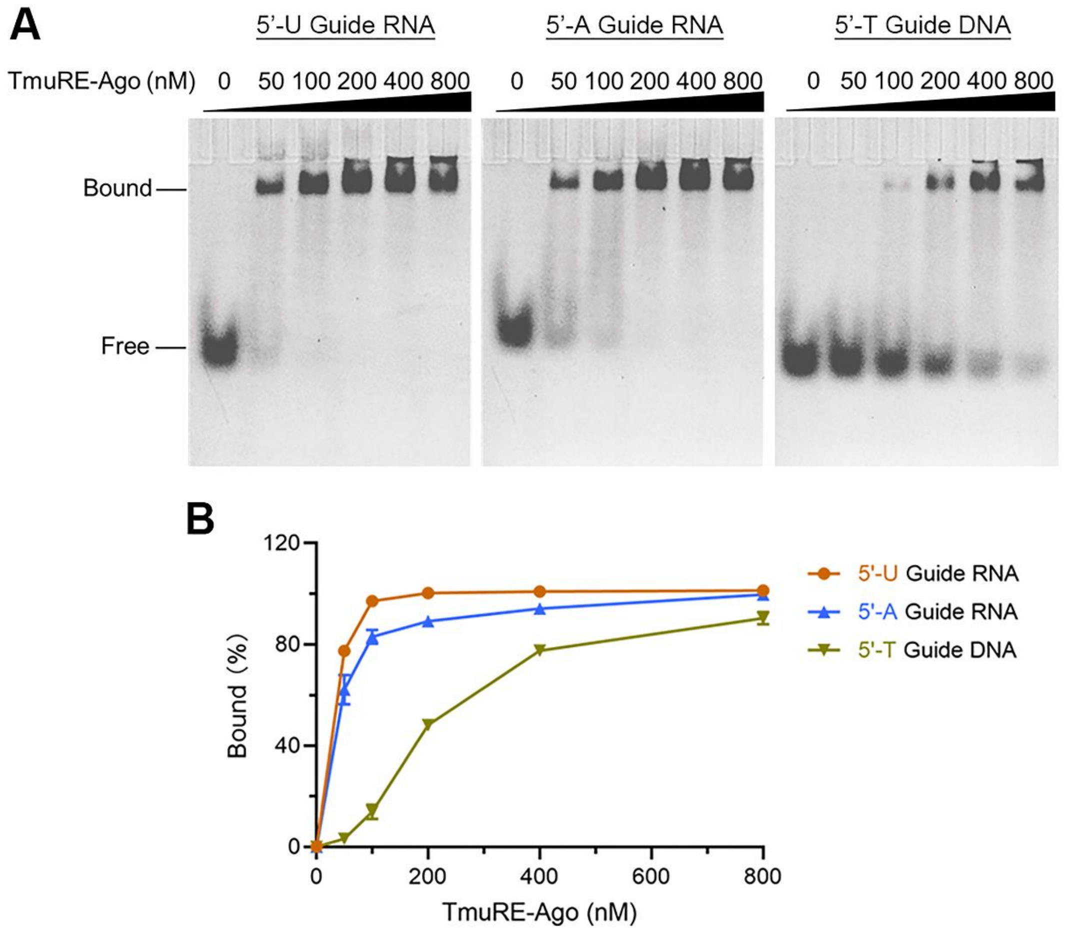

Figure S6. The binding affinity of TmuRE-Ago complex to different guide nucleic acids. (A) Guide binding assay of the TmuRE-Ago complex. 50 nM of guides (5'-U Guide RNA, 5'-A Guide RNA, and 5'-T Guide DNA) were incubated with indicated concentrations of TmuRE-Ago complex in a 10 µl mixture containing 20 mM Tris-Ac pH 7.9, 50 mM KAc, 10 mM $Mg(Ac)_2$, and 2 mM DTT at 37°C for 20 min. The reaction samples were analyzed by 8% polyacrylamide gel. (B) Quantification of guide binding from (A). The data represent the means of two independent replicates.

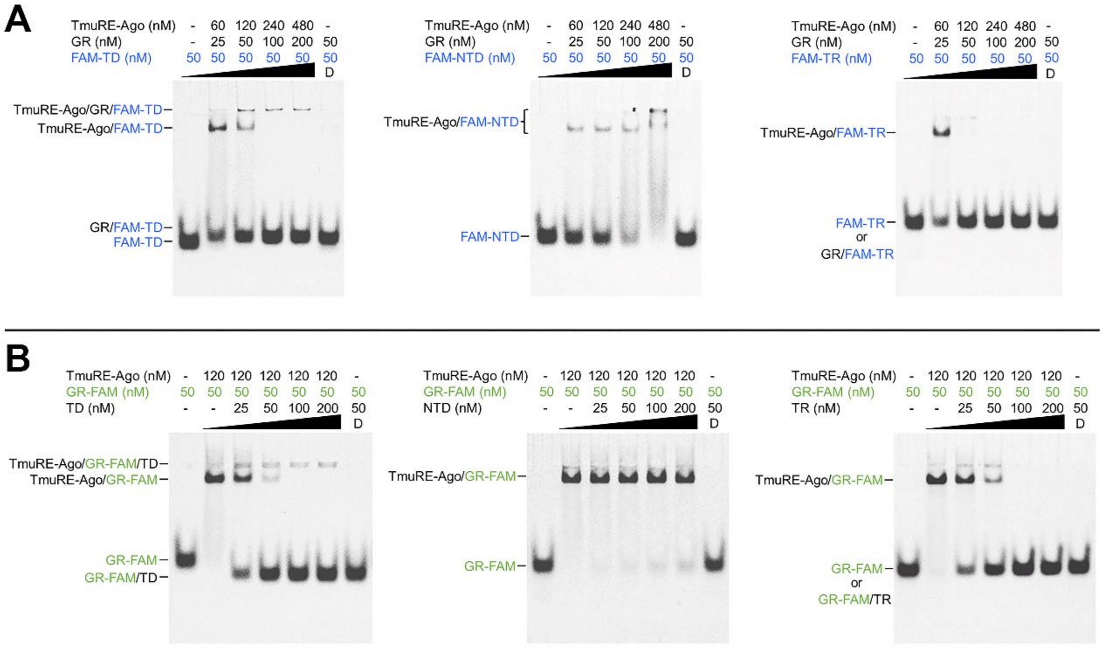

Figure S7. **Target binding of TmuRE-Ago complex pre-loaded with guide RNA.** (A) TmuRE-Ago complex at a concentration of 480 nM was initially incubated with 200 nM of 21-nt guide RNA at 37°C for 15 min to allow for guide RNA loading. Subsequently, the TmuRE-Ago complex was diluted to 60, 120, and 240 nM, and these dilutions were incubated with 50 nM of 5'-FAM-labeled target DNA (FAM-TD), non-target DNA (FAM-NTD), and target RNA (FAM-TR) at 37°C for 15 min, respectively. The reactions were analyzed by an 8% polyacrylamide gel. To ensure complete loading of guide RNA onto the TmuRE-Ago complex, the molar ratio of TmuRE-Ago complex to guide RNA was maintained at 2.4:1. (B) TmuRE-Ago complex at a concentration of 120 nM was first incubated with 50 nM of 3'-FAM-labeled guide RNA (GR-FAM) at 37°C for 15 min. The mixtures were then incubated with a gradient of target DNA (TD), non-target DNA (NTD), and target RNA (TR) (25, 50, 100, and 200 nM) at 37°C for 15 min, respectively. The reactions were analyzed using an 8% polyacrylamide gel. Data are representative of two independent experiments.

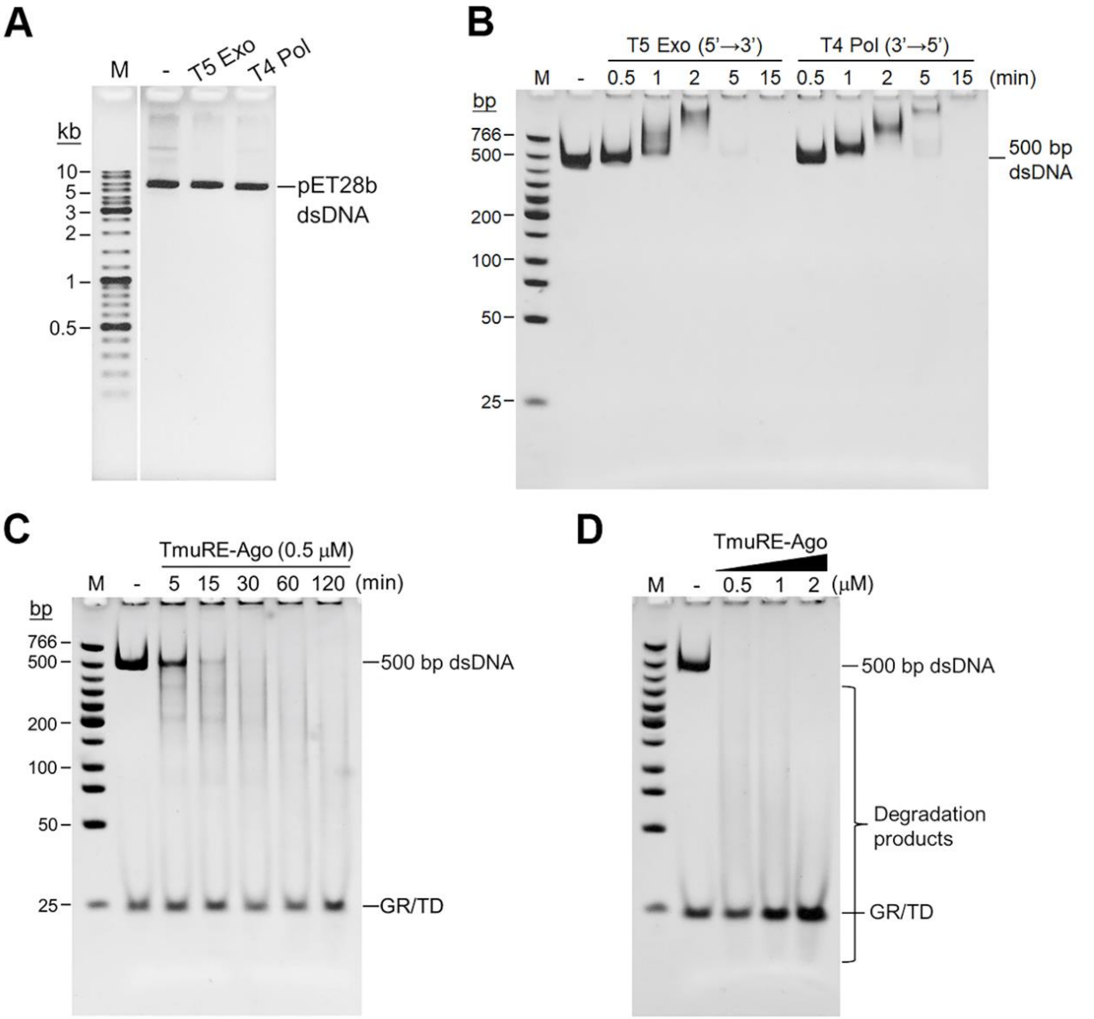

Figure S8. Evaluation of the exonuclease activity of the TmuRE-Ago complex. (A) T5 exonuclease (T5 Exo) with 5'-3' exonuclease activity and T4 polymerase (T4 Pol) with 3'-5' exonuclease activity cannot cleave circular plasmid pET28b. The reactions were conducted in the NEB CutSmart buffer (20 mM Tris-Ac, 50 mM KAc, 10 mM $Mg(Ac)_2$, 0.1 mg/ml BSA, pH 7.9), and incubated at 37°C for 15 min. The cleavage products were analyzed by 1% agarose gel. (B) Digestion of 100 ng of 500 bp DNA fragments by T5 Exo and T4 Pol for different durations. The cleavage products were analyzed by 10% polyacrylamide gel. (C) Time course of digestion of 500 bp dsDNA fragments by TmuRE-Ago complex. Reaction mixtures contained 100 ng of 500 bp DNA fragment and 500 nM TmuRE-Ago complex with guide RNA and target DNA, incubated at 37°C for the indicated times. The cleavage products were analyzed by 10% polyacrylamide gel. (D) Digestion of 100 ng of 500 bp dsDNA fragments by different concentrations of the TmuRE-Ago complex for 2 hours. The cleavage products were analyzed by 10% polyacrylamide gel.

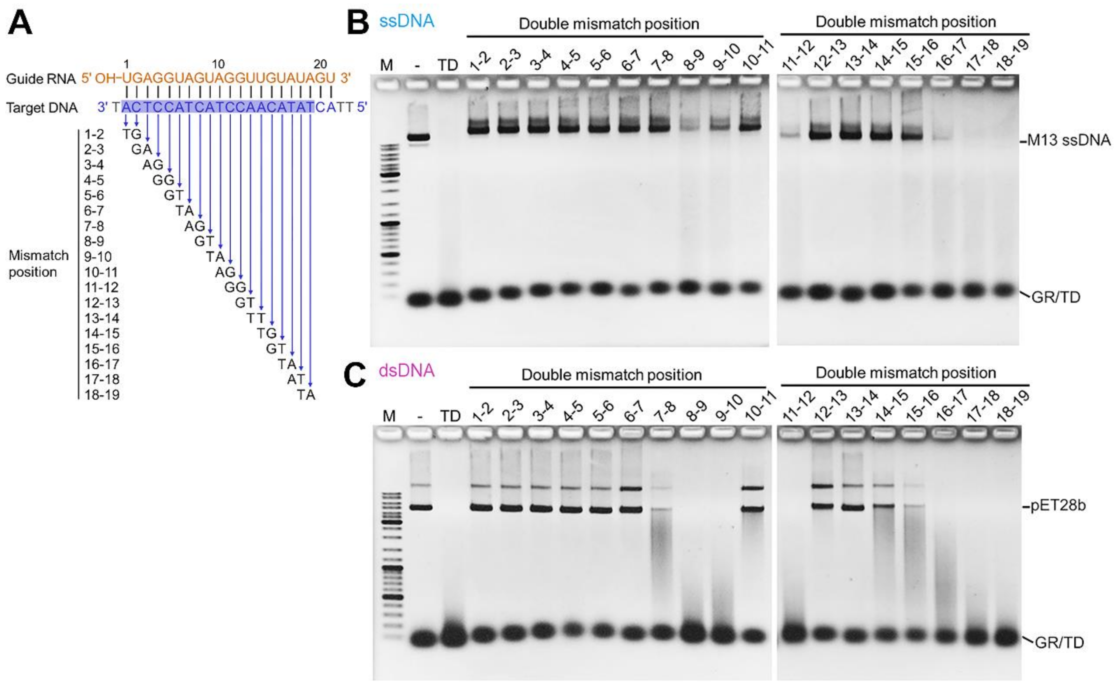

Figure S9. Effects of two consecutive nucleotide mismatches between guide RNA and target DNA on cleavage activity of TmuRE-Ago complex. (A) Schematic representation of the guide RNA and target DNAs used in (B-C). The guide sequences used were the same while the target sequences were varied to form mismatches. (B-C) Different positions of double mismatches exhibit varying effects on cleavage activities towards ssDNA (B) and dsDNA (C). Two consecutive nucleotide mismatches were introduced at positions 1-18 counting from the 5’ end of the guide RNA, and their effect on cleavage efficiency was tested. GR/TD: guide RNA/target DNA hybrids. Data are representative of two independent experiments.

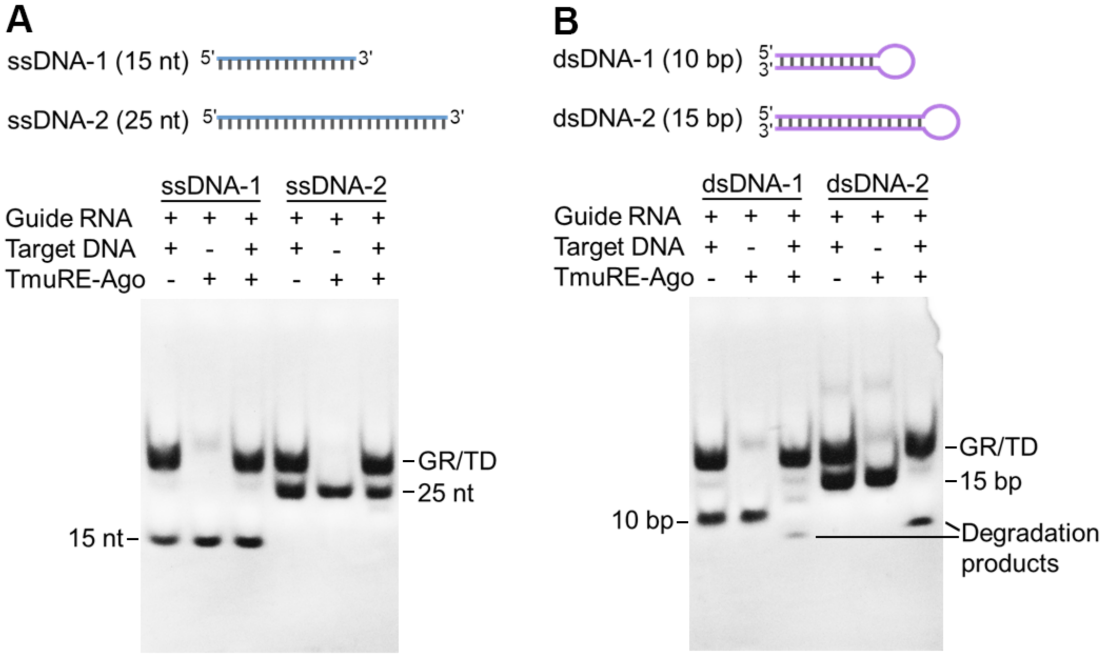

Figure S10. Analysis of short ssDNA and dsDNA cleavage by TmuRE-Ago complex. (A-B) Native polyacrylamide gel showing cleavage of ssDNAs (A) and dsDNAs (B) by TmuRE-Ago complex in the presence or absence of target DNA. The short dsDNAs originate from annealing ssDNA with two self-complementary ends.

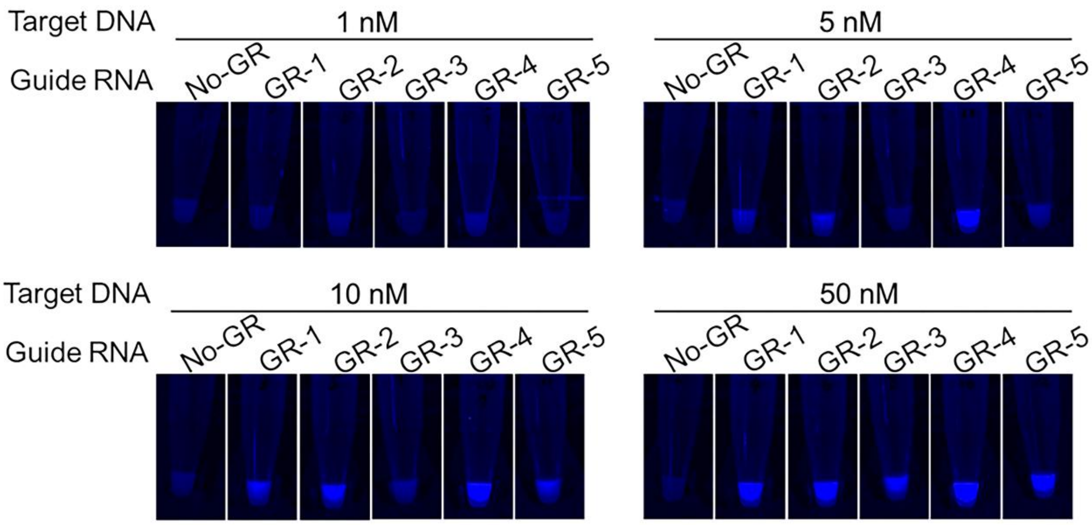

Figure S11. Fluorescence imaging detection of 100-nt target DNA by TmuRE-Ago complex with guide RNA targeting different regions of the target DNA. Related to Figure 6D.

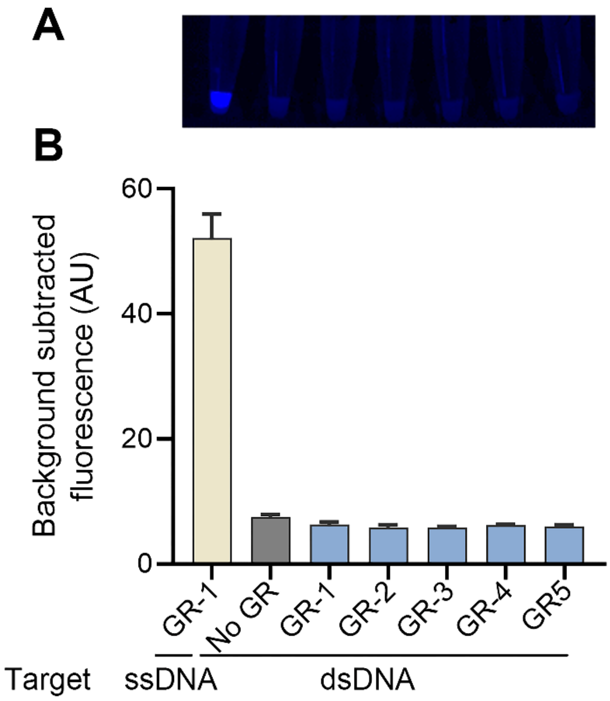

Figure S12. Investigation of dsDNA target detection by the TmuRE-Ago complex.

(A-B) End-point fluorescence photography (A) and fluorescence intensity analysis (B) following the TmuRE-Ago complex detection assays. 50 nM of the 100-bp dsDNA target was detected by TmuRE-Ago complex with different RNA guides targeting different regions of the dsDNA target. 100-nt ssDNA target was included as a positive control.

Table S1. Oligonucleotides used in this study.

Chain	Name	Sequence (5'-3')	Used in
Guide RNA	GR-21 nt	p-UGAGGUAGUAGGUUGUAUAGU	Figure S3, Figure 2E-2I
Target DNA	TD-24 nt	TTACTATACAACCTACTACCTCAT	
Guide RNA	GR-21 nt	p-UGAGGUAGUAGGUUGUAUAGU	Figure S5
Target DNA	FAM-TD-24 nt	6-FAM-TTACTATACAACCTACTAC CTCAT	
Guide	RNA-5'-P	p-UGAGGUAGUAGGUUGUAUAGU	Figure 3A-3C
	RNA-5'-OH	UGAGGUAGUAGGUUGUAUAGU	
	DNA-5'-P	p-TGAGGTAGTAGGTTGTATAGT	
	DNA-5'-OH	TGAGGTAGTAGGTTGTATAGT	
Target	RNA	UUACUAUACAACCUACUACCUCAU	
	DNA	TTACTATACAACCTACTACCTCAT	
Guide RNA	5'-OH-U	UGAGGUAGUAGGUUGUAUAGU	Figure 3D-3F
	5'-OH-A	AGAGGUAGUAGGUUGUAUAGU	
	5'-OH-C	CGAGGUAGUAGGUUGUAUAGU	
	5'-OH-G	GGAGGUAGUAGGUUGUAUAGU	
Target DNA	TD-U	TTACTATACAACCTACTACCTCAT	
	TD-A	TTACTATACAACCTACTACCTCTT	
	TD-C	TTACTATACAACCTACTACCTCGT	
	TD-G	TTACTATACAACCTACTACCTCCT	
Guide RNA	5'-OH-U	UGAGGUAGUAGGUUGUAUAGU	Figure 4，Figure 6C
Target DNA	TD-24 nt	TTACTATACAACCTACTACCTCAT	
	1	TTACTATACAACCTACTACCTCTT	
	2	TTACTATACAACCTACTACCTGAT	
	3	TTACTATACAACCTACTACCACAT	
	4	TTACTATACAACCTACTACGTCAT	
	5	TTACTATACAACCTACTAGCTCAT	
	6	TTACTATACAACCTACTTCCTCAT	
	7	TTACTATACAACCTACAACCTCAT	
	8	TTACTATACAACCTAGTACCTCAT	
	9	TTACTATACAACCTTCTACCTCAT	
	10	TTACTATACAACCAACTACCTCAT	
	11	TTACTATACAACGTACTACCTCAT	
	12	TTACTATACAAGCTACTACCTCAT	
	13	TTACTATACATCCTACTACCTCAT	
	14	TTACTATACTACCTACTACCTCAT	
	15	TTACTATAGAACCTACTACCTCAT	
	16	TTACTATTCAACCTACTACCTCAT	
	17	TTACTAAACAACCTACTACCTCAT	

	18	TTACTTTACAACCTACTACCTCAT	
Guide RNA	5’-OH-U	UGAGGUAGUAGGUUGUAUAGU	Figure S9
Target DNA	TD	TTACTATACAACCTACTACCTCAT	
	1-2	TTACTATACAACCTACTACCTGTT	
	2-3	TTACTATACAACCTACTACCAGAT	
	3-4	TTACTATACAACCTACTACGACAT	
	4-5	TTACTATACAACCTACTAGGTCAT	
	5-6	TTACTATACAACCTACTTGCTCAT	
	6-7	TTACTATACAACCTACATCCTCAT	
	7-8	TTACTATACAACCTAGAACCTCAT	
	8-9	TTACTATACAACCTTGTACCTCAT	
	9-10	TTACTATACAACCATCTACCTCAT	
	10-11	TTACTATACAACGAACTACCTCAT	
	11-12	TTACTATACAAGGTACTACCTCAT	
	12-13	TTACTATACATGCTACTACCTCAT	
	13-14	TTACTATACTTCCTACTACCTCAT	
	14-15	TTACTATAGTACCTACTACCTCAT	
	15-16	TTACTATTGAACCTACTACCTCAT	
	16-17	TTACTAATCAACCTACTACCTCAT	
	17-18	TTACTTAACAACCTACTACCTCAT	
	18-19	TTACATTACAACCTACTACCTCAT	
	19-20	TTAGAATACAACCTACTACCTCAT	
	20-21	TTTGTATACAACCTACTACCTCAT	
Guide RNA	11 nt	UGAGGUAGUAG	Figure 5A-5C
	12 nt	UGAGGUAGUAGG	
	13 nt	UGAGGUAGUAGGU	
	14 nt	UGAGGUAGUAGGUU	
	15 nt	UGAGGUAGUAGGUUG	
	16 nt	UGAGGUAGUAGGUUGU	
	17 nt	UGAGGUAGUAGGUUGUA	
	18 nt	UGAGGUAGUAGGUUGUAU	
	19 nt	UGAGGUAGUAGGUUGUAUA	
	20 nt	UGAGGUAGUAGGUUGUAUAG	
	21 nt	UGAGGUAGUAGGUUGUAUAGU	
	25 nt	UGAGGUAGUAGGUUGUAUAGUAAGC	
	30 nt	UGAGGUAGUAGGUUGUAUAGUAAGC UUGGC	
	40 nt	UGAGGUAGUAGGUUGUAUAGUAAGC UUGGCACUGGCCGUC	
	11 nt	CTACTACCTCA	
	12 nt	CCTACTACCTCA	
	13 nt	ACCTACTACCTCA	
	14 nt	AACCTACTACCTCA	

Target DNA	15 nt	CAACCTACTACCTCA	
	16 nt	ACAACCTACTACCTCA	
	17 nt	TACAACCTACTACCTCA	
	18 nt	ATACAACCTACTACCTCA	
	19 nt	TATACAACCTACTACCTCA	
	20 nt	CTATACAACCTACTACCTCA	
	21 nt	ACTATACAACCTACTACCTCA	
	25 nt	GCTTACTATACAACCTACTACCTCA	
	30 nt	GCCAAGCTTACTATACAACCTACTACC TCA	
	40 nt	GACGGCCAGTGCCAAGCTTACTATAC AACCTACTACCTCA	
	45 nt	AAACGACGGCCAGTGCCAAGCTTACT ATACAACCTACTACCTCAT	
Guide RNA	21 nt	UGAGGUAGUAGGUUGUAUAGU	Figure 5D-5F
Target DNA	24 nt	TTACTATACAACCTACTACCTCAT	
	L5R5	AGCTTACTATACAACCTACTACCTCAT AAAC	
	L10R10	TGCCAAGCTTACTATACAACCTACTAC CTCATAAACGACGG	
	L15R15	GCCAGTGCCAAGCTTACTATACAACCT ACTACCTCATAAACGACGGCCAGT	
	L20R20	CGACGGCCAGTGCCAAGCTTACTATA CAACCTACTACCTCATAAACGACGGC CAGTGCCAA	
Guide	5’-U	UGAGGUAGUAGGUUGUAUAGU-6-FAM	Figure S6
	5’-A	AGAGGUAGUAGGUUGUAUAGU-6-FAM	
	5’-T	TGAGGTAGTAGGTTGTATAGT-6-FAM	
Target	TD	TTACTATACAACCTACTACCTCAT	
Guide	GR	UGAGGUAGUAGGUUGUAUAGU	Figure S7A
Target	FAM-TD	6-FAM-TTACTATACAACCTACTACCT CAT	
	FAM-NTD	6-FAM-TTTGATATGTTGGATGATGGA GTT	
	FAM-TR	6-FAM-UUACUAUACAACCUACUACC UCAU	
Guide	GR-FAM	UGAGGUAGUAGGUUGUAUAGU-6-FAM	Figure S7B
Target	TD	TTACTATACAACCTACTACCTCAT	
	NTD	TTTGATATGTTGGATGATGGAGTT	
	TR	UUACUAUACAACCUACUACCU	

		CAU	
Guide RNA	GR-1	UGAUUAGUUCCUGGUCCCCAA	Figure 6D, Figure S11 Figure S12
	GR-2	UGUUUGUAAUCAGUUCCUUGU	
	GR-3	UGCGGCCAAUGUUUGUAAUCA	
	GR-4	UGGGGGCAAAUUGUGCAAUUU	
	GR-5	UGAAGCGCUGGGGGCAAAUUG	
Target DNA	TD-100 nt	TTTTGGGGACCAGGAACTAATCAGAC AAGGAACTGATTACAAACATTGGCCG CAAATTGCACAATTTGCCCCCAGCGCT TCAGCGTTCTTCGGAATGTCG	
**Other oligonucleotides used in this study**			
Additional DNA	ssDNA-1	CGGCTCTGGTGGTGG	Figure S10
	ssDNA-2	CGGCTCTGGTGGTGGTTCTGGTGGC	
	dsDNA-1	CGGCTCTGGTATTTTTAACCAGAGCCG	
	dsDNA-2	GTTCTGAGGGTGGCGATTTTTACGCCA CCCTCAGAAC	
	dsDNA reporter	6-FAM-GTTCTGAGGGTGGCGATTTTT ACGCCACCCTCAGAAC-BHQ-1	Figure 6B-6D, Figure S11 Figure S12

References

1. Zhao, L., Bonocora, R.P., Shub, D.A. and Stoddard, B.L. (2007) The restriction fold turns to the dark side: a bacterial homing endonuclease with a PD-(D/E)-XK motif. Embo j, 26, 2432-2442.
2. Nishino, T., Komori, K., Tsuchiya, D., Ishino, Y. and Morikawa, K. (2001) Crystal structure of the archaeal holliday junction resolvase Hjc and implications for DNA recognition. Structure, 9, 197-204.
3. Zimmermann, L., Stephens, A., Nam, S.Z., Rau, D., Kübler, J., Lozajic, M., Gabler, F., Söding, J., Lupas, A.N. and Alva, V. (2018) A Completely Reimplemented MPI Bioinformatics Toolkit with a New HHpred Server at its Core. J Mol Biol, 430, 2237-2243.
